# Abdominal wall endometrioma presenting as a cystic abdominal wall mass: a case report and literature review

**DOI:** 10.1093/jscr/rjab335

**Published:** 2021-08-26

**Authors:** Tyler Davis, Paige Moudy, Lutfi Barghuthi, Mohamed Abdelgawad, Hishaam Ismael

**Affiliations:** University of Texas at Tyler, Tyler, TX, USA; Texas College of Osteopathic Medicine, Fort Worth, TX, USA; University of Texas at Tyler, Tyler, TX, USA; University of Texas at Tyler, Tyler, TX, USA; University of Texas at Tyler, Tyler, TX, USA

## Abstract

Endometriosis is classically defined as ‘the presence of endometrial glands and stroma outside of the uterine cavity and musculature’. Although it most commonly occurs in the pelvis, various extrapelvic locations have been reported in the literature. There seems to be a strong association between abdominal wall endometriomas and previous surgical scars. In female patients presenting with a cyclically painful abdominal wall mass, a high index of suspicion for endometrioma must be maintained, especially in the setting of previous gynecologic surgery. Although there may be a role for medical management of symptoms, the most definitive treatment of an abdominal wall endometrioma appears to be wide local excision with negative margins. This paper presents a 39-year-old female with an extensive gynecologic surgical history presenting with a 6 × 6 cm cyclically tender abdominal wall endometrioma treated with wide local excision.

## INTRODUCTION

Endometriosis is classically defined as ‘the presence of endometrial glands and stroma outside of the uterine cavity and musculature’. Although it most commonly occurs in the pelvis, it can rarely appear in extrapelvic locations such as the intestines, lungs, kidneys and abdominal wall. Abdominal wall endometriomas have been shown to be associated with surgical scars (especially after c-section), and in 14–26% of cases are associated with underlying pelvic endometriosis. Although the most common complaint is cyclic menstrual pain, abdominal wall endometrioma is only diagnosed pre-operatively in 20–50% of cases. The differential diagnosis often includes abdominal wall abscesses, hematomas and metastatic disease. Hormonal treatments have been shown to relieve symptoms, but the most effective treatment of abdominal wall endometrioma appears to be wide local excision with clear margins.

In this paper, we present a case of a 39-year-old female with a 6 × 6-cm abdominal wall endometrioma treated with wide local excision.

## CASE PRESENTATION

The patient is a 39-year-old African American female who presented with a 3-year history of a tender palpable right lower quadrant abdominal mass. The associated burning pain increased with menses. The patient has a surgical history of an appendectomy and four prior Caesarean sections. Six weeks prior to presentation, she underwent a total laparoscopic hysterectomy with bilateral salpingectomy, as uterine fibroids were suspected to be the potential cause of her pain. Pathology revealed multiple myometrial leiomyomas and benign fallopian tube segments with a 5-mm paratubal cyst. Since the surgery, she reported a sudden increase in size of the lesion and acute worsening of pain. Review of systems revealed only mild constipation. Physical examination demonstrated a 7 cm × 4 cm swelling in the right lower quadrant that was exquisitely tender to palpation and exacerbated by movement. Due to the mass being away from her previous trocar incision and its cyclic nature, an endometrioma was expected.

**Figure 1:**
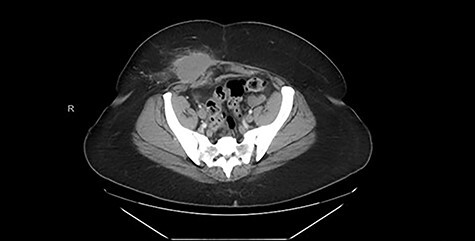
CT (axial view) demonstrating high-density soft tissue opacity measuring 5.8 × 4.4 cm in the subcutaneous tissue of the right lower quadrant abutting the anterior aspect of the abdominal wall.

Computed tomography (CT) scan of the abdomen and pelvis with contrast revealed a high-density soft tissue opacity measuring 5.8 cm × 4.4 cm in the subcutaneous tissue of the right lower quadrant abutting the anterior aspect of the abdominal wall and associated moderate surrounding subcutaneous soft tissue stranding. These findings were suggestive of a high-density seroma versus post-surgical hematoma. Figure 1 displays the pertinent CT images from admission.

**Figure 2:**
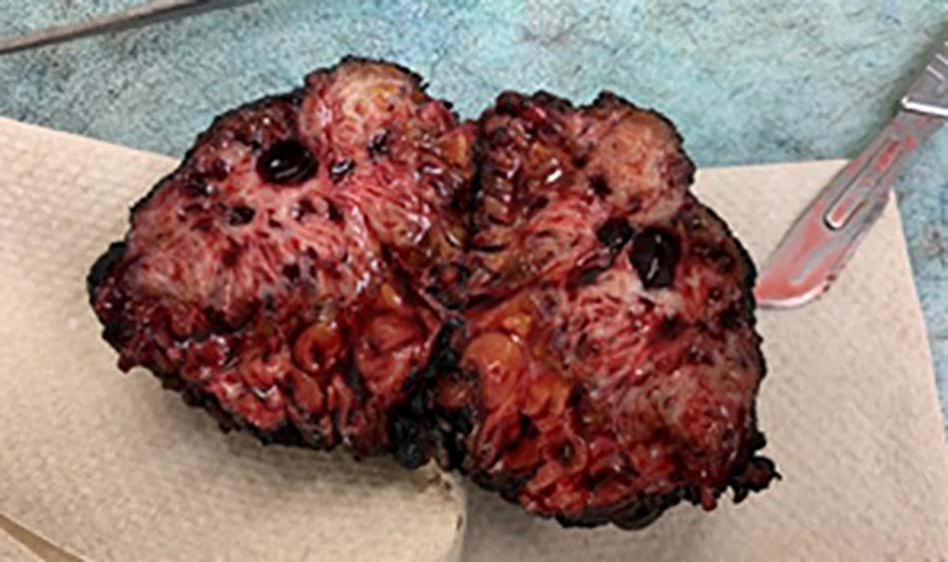
Gross cross section of the abdominal wall endometrioma.

Excisional biopsy of the anterior abdominal wall mass was performed using a transverse lower abdominal incision over the mass. Dissection was performed around the mass, maintaining a circumferential oncologic margin all the way down to the anterior rectus fascia. The posterior portion of the tumor was found to be invading the anterior rectus fascia, which was excised along with any affected muscle and delivered en bloc to the pathologist. A photograph of the 6.5 × 6.5 × 6 cm mass is shown in Fig. 2. A 15F JP drain was placed in the subcutaneous defect. The patient tolerated the procedure well and was seen in clinic 1 week later with complete resolution of pre-operative pain.

**Figure 3:**
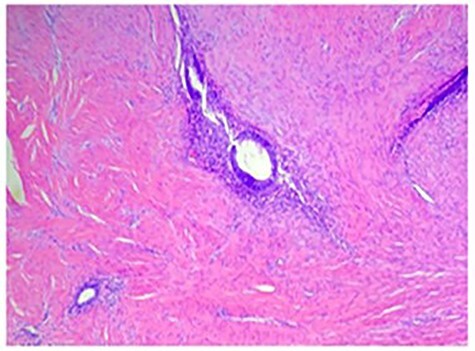
H&E stain demonstrating fibroadipose tissue with interspaced endometrial glands and stroma consistent with endometriosis.

Permanent pathologic analysis revealed fibroadipose tissue with interspaced endometrial glands and stroma, consistent with endometriosis. No endometriosis was identified at the margins. H&E stain of the mass is shown in Fig. 3.

## DISCUSSION

Large abdominal wall endometriomas are a rare occurrence. Literature review of 39 cases (summarized in Table 1) between the years of 1999 and 2020 demonstrated the average patients’ age to be 32-year old ranging from 19 to 40 years old. The average size of the endometrioma from this patient population was 3.5 × 3.1 cm [1–14]. This 39-year-old patient with an extensive history of gynecologic surgeries, had a 6.5 × 6.5 × 6 cm tumor which is within all the ranges proposed from the literature review.

**Table 1:** Literature review of 39 cases of abdominal wall endometriomas

Article title	Author(s)	Year	Patient age	Cyclical pain?	Previous gyn surgery	Tumor size (cm)	Medical management?	Surgical management?
Rare case of abdominal scar endometriosis by direct communication between fallopian tube and abdominal wall	Bartels *et al.*	2020	36	Yes	CS X1	N/A	No	Yes
Endometrioma localized in the rectus abdominis muscle: a case report and review of literature	Ozkan *et al*.	2014	31	Yes	CS x2	2 × 1.2	No	Yes
Abdominal wall endometriosis: a report of two cases	Gourgiotis *et al.*	2008	32	Yes	CS x1	3.5 × 1.5	No	Yes
			35	Yes	CS x1	4.5 × 3	No	Yes
Cesarean-section scar endometrioma: a case report and review of the literature	Kocher *et al.*	2017	37	Yes	CS x3	3.2 × 2.8	No	Yes
Abdominal wall endometriosis: a case report	Saliba *et al.*	2019	36	Yes	CS x1	3 × 3	Yes Hormonal tx w/ progestins x1 month > no improvement	Yes
			40	Yes	CS x1	N/A	Yes Analgesics + hormones (OCP + GnRH agonist) > ineffective	Yes
Endometrioma in abdominal scars: case reports of four cases and review of the literature	Uysal *et al.*	2012	27	No	Diagnostic laparoscopy	3.4 × 3.4	No	Yes
			32	No	CS x1	3.5 × 3.6	No	Yes
			28	Yes	CS x1	6.4 × 6.4	No	Yes
			30	Yes	Laparoscopic excision of ovarian endometrioma	2.3 × 2.2	No	Yes
Abdominal wall endometriomas: report of eight cases	Patterson *et al.*	1999	36	No	Diagnostic laparoscopy	N/A	No	Yes
			31	Yes	CS x2	N/A	No	Yes
			36	Yes	CS x1	N/A	No	Yes
			29	Yes	CS x1	N/A	No	Yes
			20	Yes	CS x1	N/A	No	Yes
			19	No	No	N/A	No	Yes
			28	No	CS x1, Diagnostic laparoscopy, TVH/RSO	N/A	No	Yes
			31	No	CS x1, Diagnostic laparoscopy, TVH/RSO	N/A	No	Yes
Abdominal wall endometrioma: a case report and review of the literature,	Nissotakis *et al.*	2016	24	No	CS x1	4 × 3	No	Yes
abdominal wall endometrioma: a diagnostic enigma-a case report and review of the literature	Vagholkar *et al.*	2019	29	Yes	CS x1	N/A	No	Yes
Ectopic endometriosis seeded to the rectus muscle	Ologun *et al.*	2019	40	Yes	CS x1	6.2 × 6.8	Yes Hormone tx (Lupron) x6 months	Yes
Case report: endometrioma of the abdominal wall	Huff *et al.*	2007	39	Yes	CS x1	3.5 × 3	No	Yes
Abdominal wall endometrioma	Accetta *et al.*	2011	31	Yes	CS x1	6 × 4	No	Yes
			33	Yes	CS x1	2.5 × 1.8	No	Yes
			29	Yes	CS x1	4 × 3	No	Yes
			40	Yes	No	1.5 × 1.6	No	Yes
			35	Yes	CS x1	4 × 4	No	Yes
			28	Yes	CS x1	2 × 2	No	Yes
			35	Yes	CS x1	2.3 x2.4	No	Yes
			40	Yes	CS x1	1.5 × 1.6	No	Yes
			26	Yes	CS x1	3 × 3	No	Yes
			28	Yes	CS x1	8 × 5	No	Yes
			32	Yes	CS x1	4 × 4	No	Yes
			39	Yes	CS x1	3 × 3	No	Yes
			40	Yes	CS x1	3 × 3	No	Yes
			32	Yes	CS x1	3 × 3	No	Yes
Rectus abdominal muscle endometriosis in a patient with cesarian scar: case report	Sahin *et al.*	2013	33	Yes	CS x1	2.3 × 2	No	Yes
Rectus abdominis endometrioma	Roberge *et al.*	1999	31	No	Abdominal hysterectomy	N/A	No	Yes

The most common presentation was abdominal wall mass with cyclic symptoms associated with menstruation occurring in 79.5% (31/39) of cases. Thus, a strong index of suspicion for abdominal wall endometriomas is necessary when a young to middle-age female presents with a recurrent painful abdominal wall mass.

About 94.9% (37/39) of these patients had at least one previous pelvic surgery, the most common being Cesarean-section. Based on the literature review, there seems to be a strong association between Cesarean-Section and endometriosis of the abdominal wall. Although the reflux theory is a commonly accepted mechanism of intrapelvic endometriosis, there is a similar proposed explanation of how abdominal wall endometriomas occur. It hypothesizes endometrial cells escaping through incisions in the uterus and implanting themselves within abdominal wall incision sites.

In total, 100% of patients from the literature review underwent surgical management. Only 3 patients (7.7%) underwent a trial of medical management prior to surgery. Although medical management has been previously described in the literature (particularly danazol and progesterone), it often did not result in definitive treatment. Thus, wide local excision with negative margins is becoming the accepted definitive management of this rare presentation.

## CONCLUSION

Endometriosis, particularly the extrapelvic variety, has proven to be very difficult to diagnose and is often overlooked in the world of general surgery. This suggests the importance of a thorough history and physical examination. In female patients presenting with a cyclically painful abdominal wall mass, a high index of suspicion for endometrioma must be maintained, especially in the setting of previous gynecologic surgery. Although there may be a role for medical management of symptoms, the most definitive treatment of an abdominal wall endometrioma appears to be wide local excision with negative margins.

## CONFLICT OF INTEREST STATEMENT

None declared.

## FUNDING

None.
